# Effectiveness of traditional, artificial intelligence-assisted, and virtual reality training modalities for focused cardiac ultrasound skill acquisition: a randomised controlled study

**DOI:** 10.1186/s13089-025-00469-7

**Published:** 2025-11-21

**Authors:** Yie Hui Lau, Sanchalika Acharyya, Cadence Wei Lin Wee, Huiying Xu, Rafael Pulido Saclolo, Kelly Cao, Wee Kim Fong

**Affiliations:** 1 Anaesthesiology, Intensive Care and Pain Medicine, Tan Tock Seng Hospital, Singapore, Singapore; 2 Clinical Research & Innovation Office , Tan Tock Seng Hospital, Singapore, Singapore; 3 Respiratory and Critical Care Medicine, Tan Tock Seng Hospital, Singapore, Singapore; 4 Emergency Medicine, Tan Tock Seng Hospital, Singapore, Singapore

**Keywords:** Artificial intelligence, Virtual reality, Ultrasound

## Abstract

**Background:**

Focused cardiac ultrasound (FCU) is increasingly used as an extension of physical examination to aid diagnosis and clinical decision-making. Emerging educational technologies such as artificial intelligence (AI)-enabled ultrasound devices and virtual reality (VR) simulators offer novel, cost-effective and self-directed approaches for FCU skill acquisition training. Prior studies suggest that VR-based training may be non-inferior to traditional teaching, while AI offers real-time feedback to enhance learning.

**Objective:**

This study aimed to evaluate the effectiveness and non-inferiority of AI and VR-assisted training compared to Traditional in-person instruction in achieving competency in FCU image acquisition. Secondary outcomes included time to acquire an optimal apical 4 chamber (A4C) view and self-reported confidence in image acquisition, assessed immediately post-training and at 3-month follow up.

**Methods:**

In this single-blind, randomized controlled pilot trial, 66 local medical students with no prior FCU experience were randomised into 3 arms: (1) AI-enabled ultrasound training using the Kosmos system, (2) VR-based stimulator (Vimedix), and (3) Traditional instructor-led teaching. All sessions were 60 min long. Image acquisition of 5 standard FCU views was assessed by blinded evaluators using the Rapid Assessment of Competency in Echocardiography (RACE) score at both time points.

**Results:**

Two participants were lost to follow-up (one each from the AI and VR groups). In the first assessment, the Traditional group achieved the highest mean RACE score (15.77), followed by AI (13.39) and VR (13.23). Non-inferiority testing confirmed that both AI (95% CI −∞ to 3.60; *p* < 0.001) and VR (95% CI −∞ to 3.58; *p* < 0.001) methods were non-inferior to Traditional instruction. The AI group achieved the shortest mean time to acquire an optimal A4C view (158 ± 99.1 s), followed by the VR (189 ± 94.7 s), and traditional (199 ± 115.1 s), though differences were not statistically significant (*p* = 0.591). Confidence levels were initially highest in the Traditional group, while the VR group showed higher confidence at 3-month follow-up, particularly in parasternal long-axis view acquisition.

**Conclusions:**

AI and VR-based training methods were non-inferior to traditional instruction for FCU skill acquisition. Both modalities show promise as scalable, technology-enabled alternatives in ultrasound education.

*Trial registration* This trial was registered on Clinicaltrials.gov (NCT06355557).

## Background

Focused cardiac ultrasound (FCU) allows non-sonographers to assess the heart qualitatively at the bedside using an ultrasound device. It is increasingly being used as an extension of physical examination, to aid diagnostics and guide management [1]. There is a wealth of online learning resources, but direct in-person courses and proctored hands-on training remain limited and costly. At the time of the study, one local medical school had incorporated a lecture in pre-clinical curriculum but there are no training programs existing locally. Machine learning is increasingly applied in medical imaging and deep learning algorithms are now assist novices with minimal echocardiography training to obtain diagnostic-quality images, and even help with screening for heart failure [2]. Artificial intelligence (AI) in echocardiography can enhance image acquisition by providing real-time feedback on image quality and labelling of heart structures. Ultrasound devices with built-in AI software improve real-time structure detection and identification, though more studies are needed to understand AI’s impact on learning. A prior randomised controlled trial found self-directed Virtual Reality (VR) training to be non-inferior to Traditional instruction, based on multiple-choice question and skills test and 1 month post training [3]. E-learning platforms for self-learning has also been showed to boost retention of practical FCU skills among medical students [4].

We conducted a pilot, single-blinded 3-arm randomized controlled trial to compare Traditional in-person instruction, AI-enabled handheld ultrasound, and a VR simulator for teaching novices basic FCU. AI and VR were included as comparators because they are two emerging technology-enhanced learning modalities, and both represent current directions in medical education to address resource limitations, faculty availability and learner scalability. AI-assisted ultrasound refers to systems that embed real-time image interpretation, automatic anatomical labelling and feedback on probe positioning and image quality during live scanning. These provide real-time feedback to guide the learner and reinforce correct image acquisition techniques. VR-assisted training refers to the use of high-fidelity, immersive, computer generated images that simulate probe manipulation on a mannequin or live patient. Learners can receive real time feedback on accuracy and alternative 2D anatomical images. Both modalities were selected because they are commercially available and already adopted in clinical and academic centres. We hypothesised that AI-enabled self-directed learning, coupled with e-learning, would be non-inferior to Traditional in-person training for teaching image acquisition.

## Methods

### Trial design

This was a single-centre, single-blinded, parallel-group, randomised controlled pilot trial with three arms comparing AI-based and VR-based FCU training methods to traditional in-person instruction for achieving competence in FCU. Ethics approval was provided by the National Healthcare Group Domain Specific Review Board (NHG DSRB 2023/00640). The trial was pre-registered at www.clinicaltrials.gov (NCT 06355557) and funded by Ng Teng Fong Health Innovation Program FY 2023 Pitch-for-Fund grant.

### Participants and setting

A total of 66 local medical students were recruited for this study. Medical students were selected because they were unlikely to have received further FCU training or practice during the duration of the study, compared to practicing doctors. This ensures that they remained true novices for the duration of the trial. Inclusion criteria were local medical students with no prior FCU training and adults age 21 years old and above. Exclusion criteria included age less than 21 years in 2024, prior attendance of a critical care echocardiography course or unwillingness to participate in both days of the study. Recruitment occurred in June 2024 via emails from pre-professional education office. All participants provided written informed consent prior to enrolment. Participants were randomly assigned in a 1:1:1 ratio to one of three study arms using block randomization with a fixed block size of six. The randomization sequence was generated using the ralloc package in Stata version 16 (StataCorp LLC, College Station, TX). Allocation concealment was maintained through sealed, opaque envelopes prepared by an independent coordinator not involved in study recruitment or outcome assessment. Envelopes were opened at the time of group assignment.

### Interventions/trainings

Pre-reading material was provided to all participants 1 week prior to the first training session. The pre-reading material comprised of a series of videos on basic ultrasound probe handling and instructional videos on how to obtain the 5 standard FCU views: parasternal long axis (PLAX), parasternal short axis (PSAX, mid papillary-level), apical 4 chamber (A4C), subcostal (SC), and inferior vena cava long axis (IVC) view. The participants underwent an hour of one-to-one hands-on training for image acquisition in the modality they were randomised to.

The AI-enabled system is a commercially available handheld ultrasound system for point-of-care echocardiography (Kosmos, EchoNous) (Annex, Figs. 3 and 4) which provided real-time feedback about the quality of the standard view, how to optimise the ultrasound position, and also labelling of anatomy of the heart, on the ultrasound image. This system was chosen because it is one of the only commercially available systems that provides real-time anatomical annotation and feedback on image quality improvemen. The clinical specialist for the device from will be present to help with knobology but does not provide FCU instruction. All FCU image acquisition and optimisation was self directed by the learner.

The virtual reality (VR)-simulator Vimedix (CAE^®^, now Elevate Healthcare^®^) (Annex, Figs. 5 and 6) included an echocardiography software and mannequin. It provided 2D guidance, graphics and augmented reality to show the anatomy of the heart, which was used as feedback and optimise the ultrasound probe position to get the standard images required. Setting up of the VR goggles and navigating the computer system was supported by the company representative with no FCU knowledge. All FCU image acquisition and optimisation was self directed by the learner.

This has been added to the The control arm comprised of Traditional instruction by an experienced trainer who used an ultrasound system without AI capabilities, and directly instructed the participant on basic FCU ultrasound image acquisition. The ultrasound systems used Sonosite Edge or Edge II, were different from the systems used in the assessment (Sonosite SII) to avoid bias.

### Outcome measures and assessments

Following the 1-h training session, participants underwent their first assessment. Each participant independently scanned a simulated patient using a Sonosite SII portable ultrasound machine to acquire the 5 standard FCU views. All simulated patients were healthy volunteers with good echocardiography windows. The participants were given a maximum of 30 min to complete all 5 views.

During the assessment, a study team member, blinded to group allocation and not involved in training, recorded the time required to acquire the A4C view. Timing began when the ultrasound probe first contacted the patient and ended when the participant pressed the knob to acquire the cine loop. No feedback or guidance on image acquisition or optimisation was permitted during the assessment. Immediately after scanning, participants completed a questionnaire assessing their confidence in acquiring each of the five FCU views using a standardized 3-point Likert scale (3-I’m sure of this, 2-I’m partially sure, 1-I’m not sure).

At 3-month follow-up, participants returned for assessment using the same ultrasound system and simulated patient setup. They again acquired the same 5 standard FCU views independently, and the time to A4C acquisition and confidence ratings were recorded following the same procedures. Between the first and follow-up assessments, participants did not have any additional training on FCU.

All cine clips from both time points were evaluated offline independently by 2 blinded study team members who are experienced FCU practitioners (referred to as “raters” hereafter). Image quality for each view was graded using the Rapid Assessment of Competency in Echocardiography (RACE) Score [5], a validated tool for assessing focused cardiac ultrasound proficiency. Briefly, it consists of two key components: the first evaluates image acquisition, rating the quality of 5 essential FCU views using a 6-point scale and the second focuses on image interpretation, employing a binary pass/fail system to determine whether the captured images enable clinicians to make accurate assessments of four fundamental cardiac parameters: left and right ventricular function, pericardial effusion presence, and volume status.

The primary outcome was the total RACE score in the image generation domain, reflecting the quality of captured images. Secondary outcomes included the proportion of participants achieving a pass rating in the image interpretation domain of the RACE tool, time to acquire the A4C image, participants’ self-reported confidence in image acquisition, and changes in these measures at 3-months follow-up assessment from first assessment to evaluate skill retention.

### Sample size

Sample size was calculated to assess non-inferiority of AI- and VR-based training methods compared to Traditional instruction, based on mean total RACE scores. Using a one-sided significance level of 0.05, 80% power, a standard deviation of 6.5 in total RACE score [6], and a non-inferiority margin of 5 points, the required sample size was 22 participants per group (Sample size calculator). The non-inferiority margin of 5 was chosen because it would render a change in the rank of the image quality by 1 level. This allows for two pairwise non-inferiority comparisons (AI vs. Traditional, VR vs. Traditional). The total planned sample size was 66 participants.

### Statistical methods

Descriptive statistics were used to summarize baseline characteristics and outcomes. Continuous variables were reported as mean with standard deviation (SD) or median with interquartile range (IQR) where appropriate. Group comparisons for continuous variables were conducted using one-way ANOVA, followed by post hoc pairwise comparisons with Bonferroni correction where applicable. Categorical variables were expressed as counts and percentages and were compared using chi-squared tests between groups. For the primary outcome, total RACE scores were calculated by first averaging the two raters’ assessments for each of the five cardiac views, then summing these averaged scores. One-sided t-tests were used to test for non-inferiority of AI and VR groups compared to the Traditional group. Non-inferiority was concluded if the upper bound of the 95% one-sided confidence interval for the mean difference (Traditional—Intervention) was less than the pre-specified margin of 5 points. All other outcomes were compared using appropriate univariate tests in the superiority setting. Responses to individual questions for confidence assessment were coded as either not confident (response 1 or 2) or confident (response 3) and proportions were compared between groups using chi-squared test. For each participant, the change score was calculated as follow-up score minus first assessment score and mean change scores were compared between groups using one-way ANOVA. Within-group changes in RACE total score were tested using paired t tests. A p-value of < 0.05 was considered statistically significant. Missing data were handled using complete case analysis. All analysis was performed in R 4.4.1 Jamovi 2.3.28.

## Results

The overall study sample consisted of 66 participants, equally randomized among three groups: AI (*n* = 22), Traditional (*n* = 22), and VR (*n* = 22). All participants were 21 to 25 years of age. Analysis of participant characteristics revealed no significant differences among the three intervention groups (Table 1). Gender distribution varied across groups, with the VR group having the highest proportion of female participants (68.2%), followed by the Traditional group (50%), and the AI group (36.4%). Prior experience with ultrasound machines was limited across all groups, with the VR group having the highest proportion of participants with prior experience (36.4%), followed by the Traditional group (31.8%), and the AI group (18.2%). However, statistical analysis revealed no significant differences among the groups in terms of trust in AI (*p* = 0.396), gender distribution (*p* = 0.124), or prior ultrasound experience (*p* = 0.485).

**Table 1:** Distribution of demographic characteristics

	Intervention group						*p* val*
	AI-based training model (*n* = 22)		Traditional training model (*n* = 22)		VR-based training model (*n* = 22)		
	*n*	%	*n*	%	*n*	%	
Trust in AI?							0.396
Yes	5	22.7	6	27.3	2	9.1	
Somewhat	13	59.1	9	40.9	13	59.1	
No	4	18.2	7	31.8	7	31.8	
Gender							0.124
Female	8	36.4	11	50	15	68.2	
Male	14	63.6	11	50	7	31.8	
Prior use of ultrasound machines	4	18.2	7	31.8	8	36.4	0.485

*p values are from chi-squared tests

**Fig. 1:**
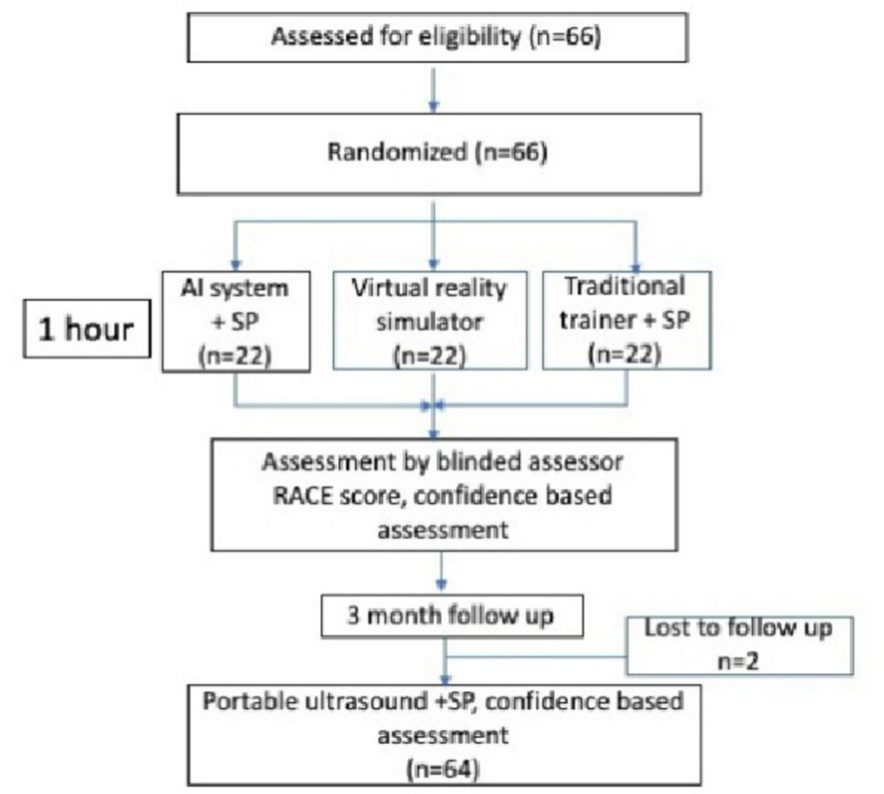
Consort flow diagram. *AI* artificial intelligence, *RACE* rapid assessment in Competency in Echocardiography, *SP* standardized patient

All 66 trainees completed the scans on the first day (first assessment). However, for the follow-up scans at 3-month (follow-up assessment), two trainees were unable to participate (Fig. 1). These absent trainees had been originally randomized to the AI and VR groups.

### Performance assessment using RACE scale

Image acquisition domain: All participants successfully obtained images for each required view, with no instances of score 0 recorded in both first and follow-up assessments.

In the first assessment, the Traditional group achieved the highest mean total RACE score of 15.77 (SD 1.89) out of a maximum of 25. The AI-assisted group had a mean score of 13.39 (SD 2.79), while the VR-assisted group showed a similar performance with a mean score of 13.23 (SD 2.18). Non-inferiority testing with a pre-determined margin of 5 points demonstrated that both AI (95% CI −inf, 3.60, *p* < 0.001) and VR-based (95% CI −inf, 3.58, *p* < 0.001) training models were non-inferior to Traditional teaching method.

At 3-month follow-up assessment, the Traditional group maintained the highest mean score of 14.89 (SD 3.13, *n* = 22), followed by VR group (mean 13.52, SD 2.14, *n* = 21), and the AI group (mean 13.24, SD 3.38, *n* = 21). These differences across the groups were not statistically significant (one-way ANOVA, *p* = 0.186).

**Fig. 2:**
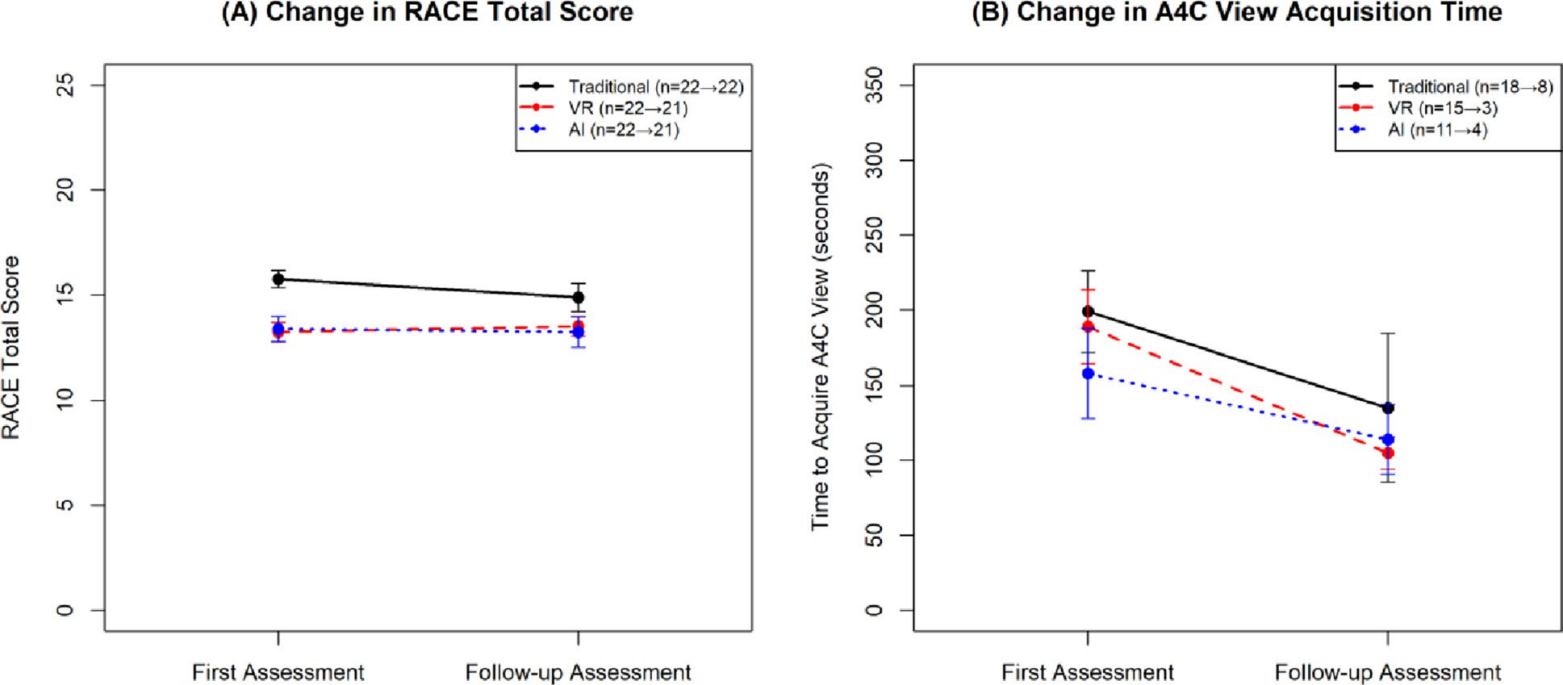
Echocardiography performance changes across training groups. **A** RACE total scores and **B** A4C view acquisition times from first to follow-up assessment. Data shown as means ± standard error. Sample sizes indicated in parentheses (first → follow-up assessment). Traditional (black solid), VR (red dashed), and AI (blue dotted) training groups

**Fig. 3:**
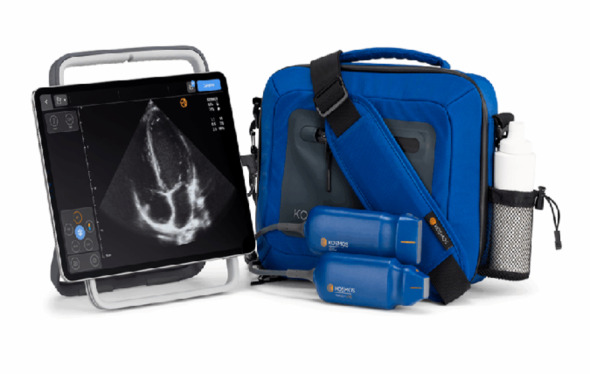
AI-enabled portable ultrasound Kosmos (Echonous) https://echonous.com/product/kosmos-ultraportable-ultrasound/

**Fig. 4:**
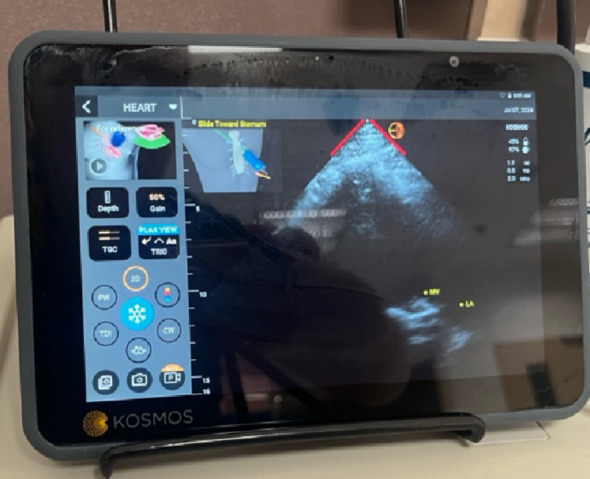
AI-enabled ultrasound system Kosmos (Echonous) showing anatomical annotations, image optimization tips and feedback about image accuracy

**Fig. 5:**
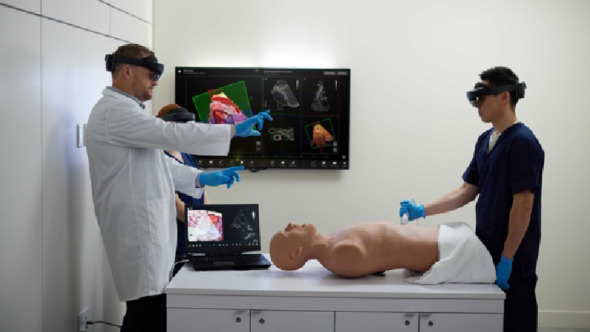
Virtual reality -assisted simulator and goggles Vimedix (Elevate Healthcare) https://elevatehealth.net/product/vimedix/

**Fig. 6:**
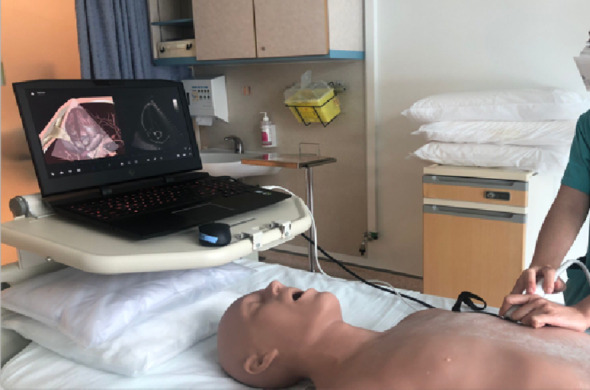
Vimedix VR-assisted simulator in use with concurrent echocardiography and real-time 2D anatomical guidance

The mean change in RACE total score at follow-up assessment from first assessment varied across the three intervention groups (Fig. 2A) although the differences across groups weren’t statistically significant (one-way ANOVA *p* = 0.483). The Traditional group showed the largest decrease (mean − 0.88, SD 3.10, paired t-test *p* = 0.194), while AI group showed minimal change (mean − 0.07, SD 2.75, paired t-test *p* = 0.907). The VR group demonstrated a slight increase (mean 0.24, SD 3.19, paired t-test *p* = 0.736).

Image interpretation domain: We employed a conservative approach to determine pass/fail status based on the raters’ assessments of overall image quality for left ventricular (LV) function, right ventricular (RV) function, volume status, and pericardial assessments. If any of the two raters rated the overall image quality permitting meaningful interpretation across all four tasks, then the status will be pass, otherwise fail. This binary classification method prioritized recognition of minimal competency, as indicated by any rater’s assessment of acceptable image acquisition skills across all 4 tasks.

Analysis of initial competency in the first assessment revealed varying pass rates across training groups. In the first assessment, the Traditional group demonstrated the highest pass rate (81.8%, 18/22), followed by the VR group (72.7%, 16/22), while the AI group showed a notably lower pass rate (50.0%, 11/22). The difference in pass rate between AI and Traditional groups was statistically significant (chi-squared test *p* = 0.026), whereas there was no evidence of a statistically significant difference between VR and Traditional groups (*p* = 0.472). However, in the follow-up assessment, all groups showed comparatively lower pass rates than the first assessment. While the Traditional group maintained the highest pass rate in the follow-up assessment (36.4%, 8/22), the differences were not statistically significant when compared to either the AI group (19.0%, chi-squared test *p* = 0.206) or the VR group (14.3%, chi-squared test *p* = 0.097).

### Time taken to acquire apical 4 chamber view

We assessed the time to measure A4C views only if the image was of adequate quality. Among the 45 participants with Pass rank in the first assessment, 44 had data collected on time taken to acquire the A4C view. Analysis of this time variable showed varying performance across groups (Fig. 2B). The AI group demonstrated the shortest mean acquisition time (158 ± 99.1 s, *n* = 11), followed by the VR group (189 ± 94.7 s, *n* = 15), while the Traditional group took the longest (199 ± 115.1 s, *n* = 18). These differences were not statistically significant (one-way ANOVA *p* = 0.591). However, the considerable standard deviations in all groups suggest high variability in individual performance, with the Traditional group showing the highest variability (SD = 115.1 s).

In the follow-up assessment, while the sample sizes were notably smaller, all groups showed faster acquisition times: VR group achieved the fastest time (105 ± 18.6 s, *n* = 3), followed by AI group (114 ± 46.6 s, *n* = 4), and Traditional group (135 ± 140.0 s, *n* = 8), though the differences were not statistically significant (one-way ANOVA *p* = 0.829).

The substantial reduction in participant numbers with passing grades in the follow-up assessment (from 44 to 15 total) should be noted when interpreting these results.

### Self-reported confidence-based assessments

Table 2 presents the number and percentage of trainees who reported feeling confident about their image acquisition abilities across five different echocardiographic views, comparing three training groups at both first and follow-up assessments.

In the first assessment, the Traditional group had the highest proportion of trainees feeling confident about their image acquisition skills across most views, with 54.5% feeling confident in parasternal long axis (PLAX), 50% in parasternal short axis (PSAX), and 50% in IVC views. The only statistically significant difference between groups was found in IVC view acquisition, where significantly fewer trainees felt confident in the VR group (13.6%, chi-squared test *p* = 0.010) compared to Traditional. By the follow-up assessment, there was a notable shift in confidence distribution. The VR group showed marked improvement in PLAX view acquisition confidence, with 61.9% of trainees reporting confidence (compared to Traditional group’s 50%, chi-squared test *p* = 0.432). Confidence in apical 4-chamber and subcostal views remained relatively lower across all groups throughout both assessments, with the proportion of confident trainees ranging from 9.1 to 36.4%, and no significant differences between groups (all p-values > 0.05).

**Table 2:** Confidence-based assessment: count and percentage of trainees reporting “I’m sure of this” in image acquisition confidence across training methods

View	First assessment			Follow-up assessment		
	Traditional	AI	VR	Traditional	AI	VR
PLAX	12 (54.5)	9 (40.9) (*p* = 0.365)	8 (36.4) (*p* = 0.226)	11 (50)	9 (42.9) (*p* = 0.639)	13 (61.9) (*p* = 0.432)
PSAX	11 (50)	5 (22.7) (*p* = 0.060)	7 (31.8) (*p* = 0.220)	12 (54.5)	8 (38.1) (*p* = 0.280)	12 (57.1) (*p* = 0.864)
A4C	8 (36.4)	6 (27.3) (*p* = 0.517)	3 (13.6) (*p* = 0.082)	7 (31.8)	6 (28.6) (*p* = 0.817)	5 (23.8) (*p* = 0.558)
Subcostal	6 (27.3)	2 (9.1) (*p* = 0.118)	3 (13.6) (*p* = 0.262)	3 (13.6)	4 (19) (*p* = 0.631)	3 (14.3) (*p* = 0.951)
IVC	11 (50)	5 (22.7) (*p* = 0.060)	3 (13.6) (*p* = 0.010)	6 (27.3)	6 (28.6) (*p* = 0.924)	4 (19) (*p* = 0.523)

p values are from chi-squared tests

## Discussion

Based on this comprehensive analysis of AI-based, Traditional, and VR-based echocardiography training methods, several key insights emerge. While the Traditional method maintained generally higher performance metrics in the first assessment, particularly in pass rates (81.8%) and trainee confidence levels, both alternative methods showed promising aspects. The VR group demonstrated the highest overall improvement rate in RACE scores (57.1% vs. 40.9% in Traditional). The AI group, while showing lower initial pass rates (50.0%), achieved the fastest image acquisition times for the apical 4-chamber view (158 ± 99.1 s vs. 199 ± 115.1 s in Traditional). This was especially noticeable immediately after training, although it did not reach statistical significance. Self-reported confidence levels evolved differently across groups, with the VR group showing marked improvement in parasternal views by the follow-up assessment (reaching 59.1% for PLAX), while the AI group demonstrated consistent, modest improvements across most views. It’s important to note that these percentages represent only those trainees who felt fully confident (“Sure”) in their image acquisition skills, and do not account for those who felt “Partially Sure”.

However, the general decline in pass rates during the follow-up assessment across all groups (Traditional: 36.4%, AI: 18.2%, VR: 13.6%) suggests that skill retention remains a challenge regardless of training method. In terms of confidence-based assessment, participants trained in the Traditional arm tended to report more confidence in FCU scans. The reduction in acquisition times for the apical 4 chamber view at follow-up likely reflects a practice effect, as participants became more familiar with the imaging procedure despite no additional training being provided during the interim period.

Strengths: This is the first 3-arm study comparing a novel method of Focused cardiac ultrasound instruction targeting image acquisition skills alone. This is driven by the need to allow efficient, cost-effective training of large numbers of new-learners. For our study, all ultrasound clips were reviewed by two independent assessors with the RACE scores averaged, reducing inter-observer variance. The study also shows.

Limitations of the study included that the study did not account for attrition of knowledge and on-going training required to maintain skill competency within 3 months of the study. This was pragmatic and similar to the experience of many attendees of FCU courses who do not have formal training programs following an introductory course. While it does not mirror real-world continuous learning, it allows objective evaluation of knowledge decay, a recognised challenge in FCU education. This also provides a conservative estimate of the effectiveness of the various interventions for true novices. The AI and VR systems are not commonly encountered and it was likely that participants randomized to those groups required time to familiarize themselves with the systems. This may have reduced the effective time that they had for hands-on practice. All participants were sent introduction videos for the AI and VR systems as part of their pre-reading material to maximize familiarity prior to their hands-on session. The simulated patients were all healthy volunteers with good echocardiography windows, which is not always found realistically in clinical practice. The subjects were a homogeneous source which will limit generalizability, but this reflects training on real novices. “Trust in AI” was surveyed to characterize the study sample, but there was insufficient power for meaningful statistical analyses between trust levels and outcomes, due to the small sample sizes. Future studies with larger samples may want examine these associations which could influence acceptance and confidence in skill acquisition. Lastly, the 1 h hands-on training time may be too short and may underestimate the true benefits of longer hands-on practice for AI and VR- training, but it was the study was designed such for practical reasons and translates to similar individual hands-on training time for a one day course.

The use of AI system in subsequent practices was not studied and can potentially reinforce further learning without the need of an experienced FCU practitioner. Future studies should be focused on the continual learning with the aid of an AI system.

The combined use of either AI or VR with an instructor was also not studied.

The parasternal windows appear to be consistently the echocardiographic windows which novice learners were most confident in scanning for both phases of the study, consistent with practical experience.

## Conclusion

For novices, Traditional in-person training of focused cardiac ultrasound currently maintains certain advantages, Both AI-systems and VR-simulators showed promise as are self-directed alternatives, which will reduce the need for faculty. VR simulators especially are still potentially advantageous in specific aspects of FCU such has retention of parasternal long and short axis image acquisition skills, to complement to Traditional teaching. Further research with larger samples, and longer follow-up periods incorporating more practice to accrue the critical mass of scans required for competency, may be warranted to fully establish their comparative effectiveness.

## Data Availability

All data generated or analysed during this study are included in this published article [and its supplementary information files].

## Annex

See Figs. 3, 4, 5 and 6.

## Abbreviations

AI: Artificial intelligence
A4C: Apical 4 chamber
FCU: Focused cardiac ultrasound
IVC: Inferior vena cava
PLAX: Parasternal long axis
PSAX: Parasternal short axis
VR: Virtual reality
